# Acute ischemic stroke revealing an internal carotid artery dissection in a 12-year-old child: case report and literature review

**DOI:** 10.1097/MS9.0000000000002818

**Published:** 2025-01-09

**Authors:** Bouchra Chahboun, Ikram Mekkaoui, Ousmane Kaba, Yassmine Zoubaa, Ilias Laaribi, Houssam Bkiyar, Brahim Housni

**Affiliations:** a Intensive Care Unit, Mohammed VI University Hospital, Oujda, Morocco; b Faculty of Medicine and Pharmacy of Oujda, Mohamed First University, Oujda, Morocco

**Keywords:** acute stroke, internal carotid artery dissection, ischemic stroke, magnetic resonance angiography, pediatrics

## Abstract

**Introduction and importance:**

Internal carotid artery (ICA) dissection is a known cause of stroke but is often underdiagnosed in pediatric patients. Pediatric ischemic stroke significantly contributes to juvenile brain injury. Currently, there are no universally accepted guidelines for managing stroke in children.

**Case Presentation:**

We report the case of a 12-year-old girl who was admitted with sudden left hemiplegia and aphasia. Imaging revealed right carotid artery dissection and ischemic stroke in the right middle cerebral artery territory. She was managed with aspirin (75 mg/day) and enoxaparin (3000 IU/12 h) and remains under observation with persistent symptoms.

**Clinical discussion:**

This case highlights the difficulty in diagnosing ICA dissections in pediatric patients and the lack of clear guidelines for their management. The use of aspirin and enoxaparin represents an empirically-based therapeutic approach, underscoring the urgent need for further research to better understand the risk factors and pathogenic mechanisms of this condition. The persistence of symptoms in our patient also emphasizes the importance of close monitoring and individualized management.

**Conclusion:**

ICA dissection in pediatric stroke is hard to diagnose and treat. Research, empirically-based treatments, and personalized monitoring are crucial for better management and outcomes.

## Introduction

Although stroke is relatively uncommon in children, it can result in substantial morbidity and mortality. The incidence of acute ischemic stroke in the pediatric population ranges from 3 to 8 per 100,000 children annually^[1]^. Acute ischemic stroke in children is attributed to arterial dissection in up to 20% of cases^[2]^. The most common etiologic factors include congenital and acquired heart diseases, vasculopathies, infections, genetic conditions, and metabolic disorders. Other etiologic factors include cerebral vessel dissection, with carotid artery dissection (CAD) accounting for 7.5–20% of ischemic stroke cases^[3,4]^. Magnetic resonance imaging (MRI) is the primary method used to detect CAD and assess brain damage^[5]^. Herein, we present the case of a 12-year-old girl with sudden left hemiplegia and aphasia, diagnosed with a right CAD and ischemic stroke in the right middle cerebral artery territory. She is currently being treated with aspirin and enoxaparin, with persistent symptoms under observation.

## Presentation of case

We report the case of a 12-year-old girl with no significant medical history who presented with sudden onset left hemiplegia and aphasia 6 hours prior to admission to the pediatric emergency. Clinical examination revealed a conscious patient with a Glasgow Coma Scale (GCS) score of 11/15 (E4V1M6), aphasia, and left hemiplegia. She was hemodynamically stable with a blood pressure of 105/70 mmHg and a heart rate of 56 bpm. Respiratory assessment showed an SpO2 of 95%. Her temperature was 36.7°C, and her capillary blood glucose was 1.06 g/L. Cardiovascular examination revealed normal heart sounds without murmurs or additional sounds, and no signs of right heart failure. Peripheral pulses, including cervical pulses, were present and symmetrical. Systolic blood pressure ranged between 105 and 110 mmHg, and diastolic blood pressure was between 65 and 70 mmHg. The remainder of the clinical examination was unremarkable.

Biological assessment showed normal lipid profile, liver enzymes, and electrolytes. Prothrombin time was 97%, international normalized ratio was 1.01, and fibrinogen level was 2.3 g/L. Complete blood count was within normal limits.

**Figure 1.**
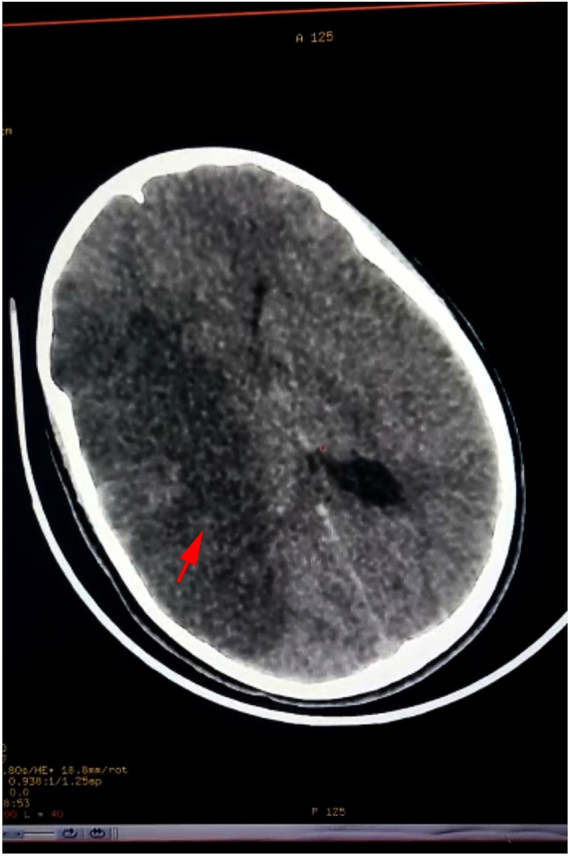
Non-contrast head CT scan revealed a spontaneous hypodensity (arrow) in the right middle cerebral artery territory.

A non-contrast head CT scan revealed a spontaneous hypodensity in the right middle cerebral artery territory, consistent with an ischemic stroke affecting the right middle cerebral artery territory (Fig. 1).

**Figure 2.**
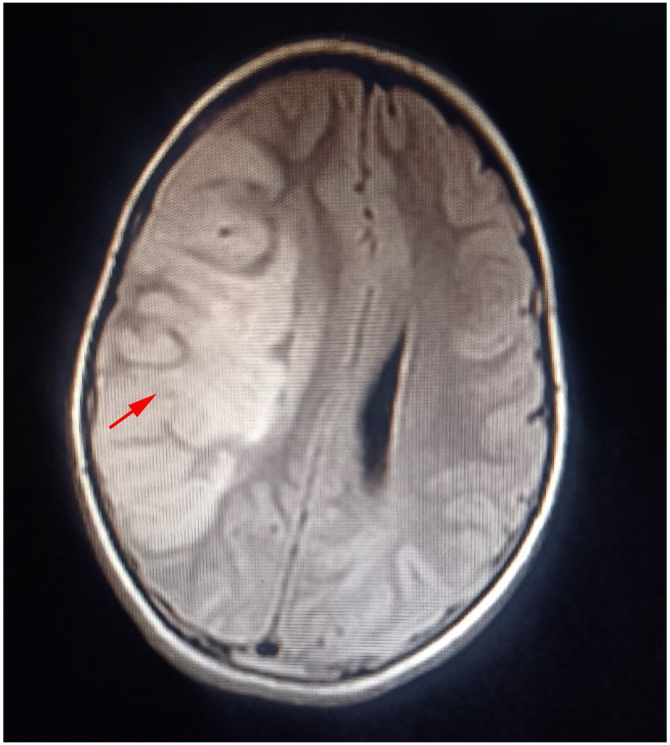
Subsequent brain MRI confirmed the presence of an ischemic stroke in the right middle cerebral artery territory due to.

A subsequent brain MRI confirmed the presence of an ischemic stroke in the right middle cerebral artery territory due to dissection of the right carotid artery with mass effect and subfalcine herniation (Fig. 2).

**Figure 3.**
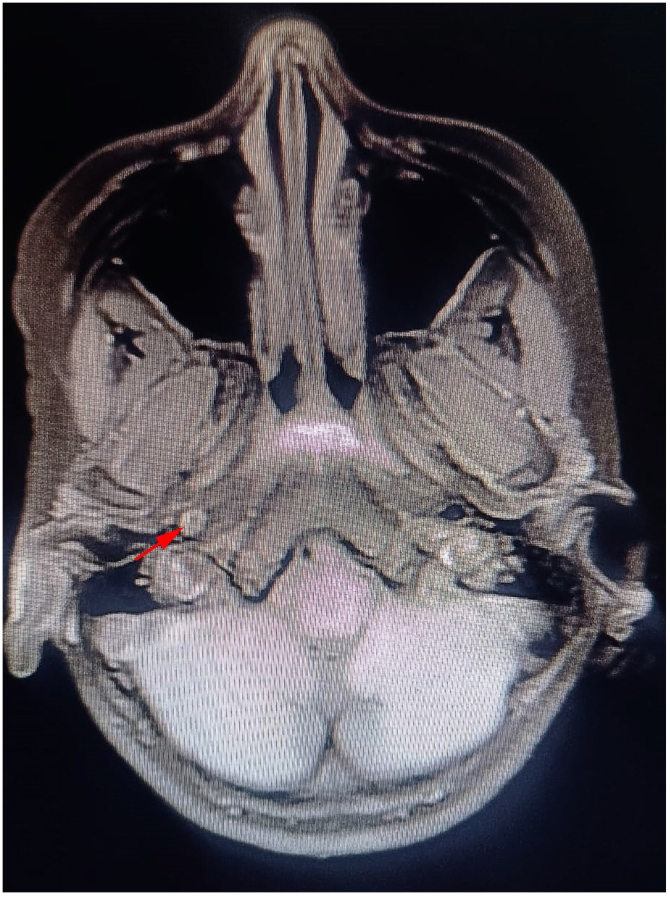
Angio-CT scan of the supra-aortic trunks showed a narrowed right internal carotid artery, measuring 2 mm in diameter.

An angio-CT scan of the supra-aortic trunks showed a narrowed right internal carotid artery (ICA), measuring 2 mm in diameter compared to the left side. The caliber decreased to 1 mm at the cavernous portion of the right ICA (compared to 4 mm on the left side). This narrowing extended from the cervical portion of the right ICA to the right middle cerebral artery, with thinning of its branches. These findings are consistent with a dissection of the right carotid artery (Fig. 3).

The patient was managed with anticoagulation therapy, including aspirin at a dose of 75 mg/day and subcutaneous enoxaparin at 3000 IU every 12 hours.

The patient remains under observation in the pediatric ICU for 2 days. Aphasia and hemiplegia persist, with a GCS score of 11/15. Cardiovascular and respiratory parameters remain stable without abnormalities then she was transferred to the pediatric department and discharged 2 weeks later, symptom-free on therapeutic enoxaparin, followed by daily aspirin therapy.

## Discussion

CAD is a leading cause of acute ischemic stroke in children. Brain and neck CADs are significant causes of cerebrovascular injuries in children^[3,4]^. The annual incidence of spontaneous CAD ranges from 2.6 to 3.0 per 100,000 individuals^[6]^. These dissections can be either spontaneous, often occurring intracranially, or traumatic, typically extracranial^[7]^. The risk factors for CAD in pediatric patients include head and neck injuries, connective tissue disorders such as Ehlers–Danlos syndrome or Marfan’s syndrome, being male^[2]^, and states of hypercoagulability^[8]^. However, as in our reported case, who is a 12-year-old girl with no history of trauma nor connective tissue disorder, it is important to note that spontaneous CAD is also frequently encountered. Causes of spontaneous, non-traumatic CAD have been identified, including infections such as varicella-zoster virus and pharyngeal infections, Moyamoya disease, arteriopathies, fibromuscular dysplasia, atherosclerosis, and cystic medial necrosis^[4]^. Additionally, some reports suggest a link between rotational changes, tolerability to G forces, and dissection, as observed in activities like water slides^[9]^, and roller coasters^[10]^.

Clinical signs associated with CAD are typically acute and include visual disturbances, nausea, vomiting, headache, hemiparesis, and ataxia^[11]^. Other possible symptoms depend on the cerebral area affected by hypoperfusion, with neurologic signs potentially appearing weeks after the trauma when the etiology is of traumatic nature^[12]^. In children, the occurrence of seizures is possible^[13]^. Our patient exhibited aphasia and hemiplegia without seizures.

Diagnosing CAD is often challenging. For patients with suspected dissection, MRI/MRA is the first-line imaging modality. This noninvasive technique does not involve radiation and can simultaneously image for both dissection and acute ischemic stroke. MRI provides a noninvasive method to detect acute ischemic stroke, offering a quicker and safer diagnostic approach^[5]^. Historically, angiography was considered the gold standard for diagnosing CAD, however, it is rarely performed in children due to the requirements for arterial vascular access, general anesthesia, and exposure to ionizing radiation. In particular, MRI can detect the intramural hematoma, which appears as a hyperintensity along the vessel wall on T1 fat-saturated imaging. In our case, angio-CT scan has enabled the diagnosis of CAD and ischemic stroke.

The treatment of ICA dissection remains a topic of debate due to the absence of controlled studies, randomized controlled trials, systematic reviews, or meta-analyses of observational data^[14]^. The effectiveness of antiplatelet agents and anticoagulation is still uncertain. Previous studies in adults suggest that thromboembolism is the main mechanism of acute ischemic stroke in CAD, recommending anticoagulation therapy for 3–6 months^[15]^. However, this treatment regimen is not supported by any prospective randomized study. The mechanisms of cerebral ischemia in intracranial artery dissection are likely similar to those in cervical artery dissection^[12]^. In a trial conducted by Markus *et al*^[16]^ involving 250 adult patients (aged 18 to 87 years) with cervical artery dissection, it was demonstrated that both antiplatelet drugs and anticoagulants had equal efficacy in preventing stroke and death. Additionally, aspirin was recommended for secondary prevention after a transient ischemic attack or ischemic stroke, with the trial data showing a 13% reduction in the long-term risk of recurrent stroke. Additionally, the use of antiplatelet therapy is also contentious. A recent randomized controlled trial did not determine whether antiplatelet therapy or anticoagulation was superior^[15]^. Antiplatelet agents (Aspirin) and anticoagulation (Enoxaparin) were the main therapeutic modalities in our reported case.

## Conclusion

CAD is a significant cause of acute ischemic stroke and cerebrovascular injuries in children, with an annual incidence of 2.6 to 3.0 per 100,000 individuals. CAD can be spontaneous or traumatic, with risk factors including head and neck injuries, connective tissue disorders, and being male. Spontaneous CAD can also result from infections, Moyamoya disease, and other conditions. Symptoms are acute, such as visual disturbances, headache, and hemiparesis. MRI is the preferred diagnostic tool due to its noninvasive nature. Treatment remains debated, with antiplatelet agents and anticoagulation showing similar efficacy in preventing stroke, but their use is not yet standardized.

## Data Availability

Data is available upon reasonable request.

## Patient’s parents perspective

The parents are satisfied with the diagnostic and therapeutic procedures.
